# Supplementary material for: SMaRT lncRNA controls translation of a G‐quadruplex‐containing mRNA antagonizing the DHX36 helicase
Source: EMBO Rep. 2020 Apr 26;21(6):e49942. doi: 10.15252/embr.201949942 (PMC7271651; doi:10.15252/embr.201949942)
Supplement: Supplementary file 1 — Expanded View Figures PDF [file EMBR-21-e49942-s001.pdf]

Expanded View Figures

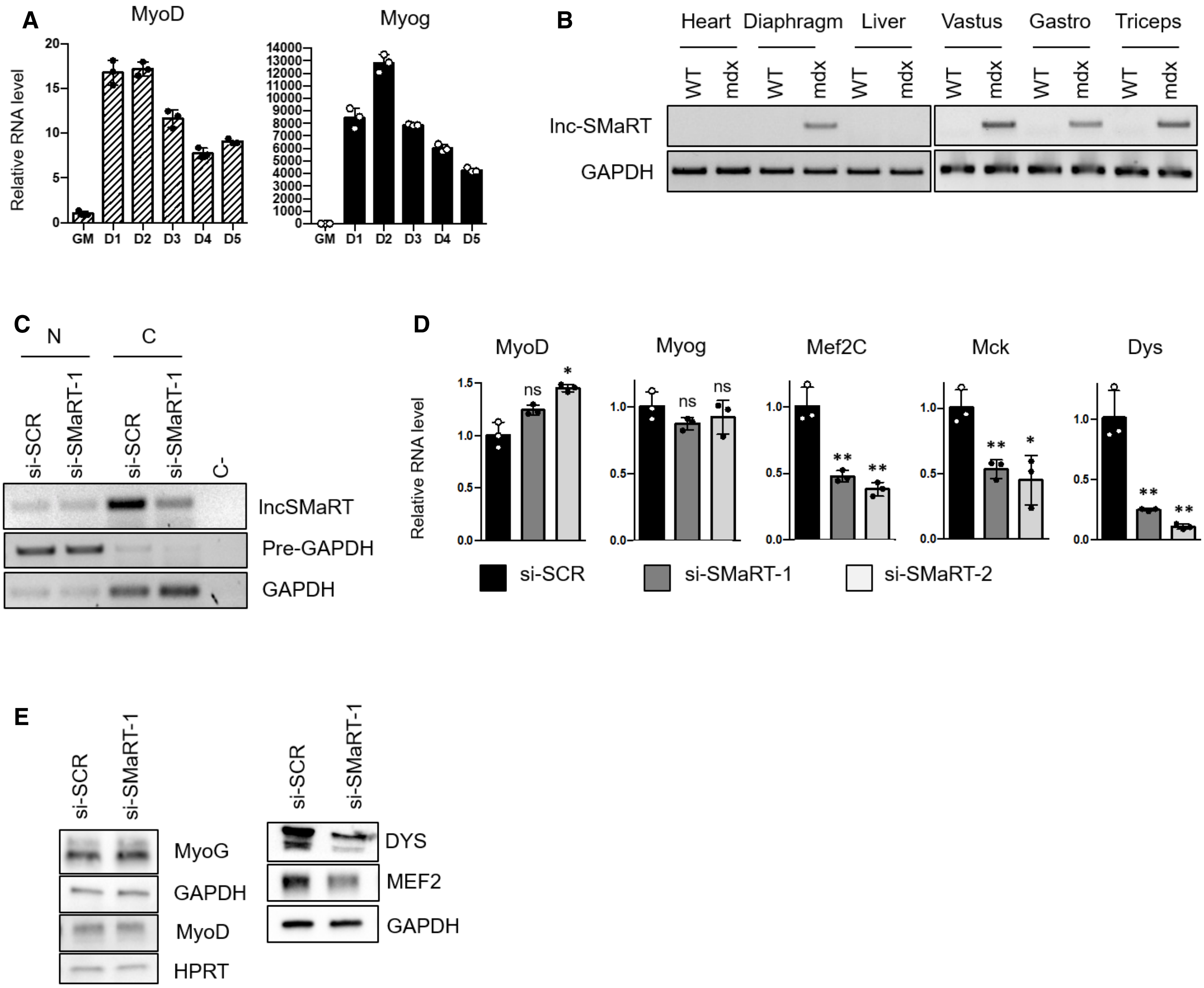

Figure EV1. Lnc-SMaRT depletion inhibits myoblast differentiation.

A qPCR analysis of MyoD and Myog RNA expression in C2C12 cells undergoing differentiation at the indicated time points. Data are presented as the mean ± s.e.m. of three biological replicates (dots) normalized against the GAPDH mRNA.

B RT–PCR showing the expression levels of lnc-SMaRT in RNA extracted from the indicated muscle tissues of control (WT) and *mdx* mice (*mdx*). GAPDH was used as control. Representative results from three independent experiments are shown.

C RT–PCR on nuclear (N) and cytoplasmic (C) extracts showing the subcellular levels of lnc-SMaRT RNA upon siRNAs treatment. RNA was isolated from C2C12 myoblasts treated with either control siRNA (si-SCR) or siRNA against lnc-SMaRT (si-SMaRT-1) and induced to differentiate for 2 days. GAPDH mRNA and pre-mRNA (pre-GAPDH) were used, respectively, as cytoplasmic and nuclear controls. Representative results from three independent experiments are shown.

D qPCR analysis of indicated muscle differentiation marker (MyoD, Myog, Mef2C, Mck, and Dys) mRNA expression in C2C12 cells undergoing differentiation (D2) in control samples (black bars) or samples depleted of lnc-SMaRT with two different siRNAs (gray and white bars). The RNA expression levels in qPCR analyses were normalized against GAPDH mRNA and expressed as relative quantities with respect to the si-SCR sample set to a value of 1. Data are presented as the mean ± s.e.m. of three biological replicates (dots). Statistical analysis was performed with analysis of variance (ANOVA) followed by Dunnett's multiple comparison test. *$P < 0.05$, **$P < 0.01$.

E Western blot on protein extract from C2C12 myoblasts treated with either control siRNA (si-SCR) or siRNA against lnc-SMaRT (si-SMaRT-1) and induced to differentiate for 2 days. MYOG, MYOD, DYS, and MEF2C antibodies were used. HPRT and GAPDH were used as loading controls. Representative results from three independent experiments are shown.

Source data are available online for this figure.

 © 2020 The Authors

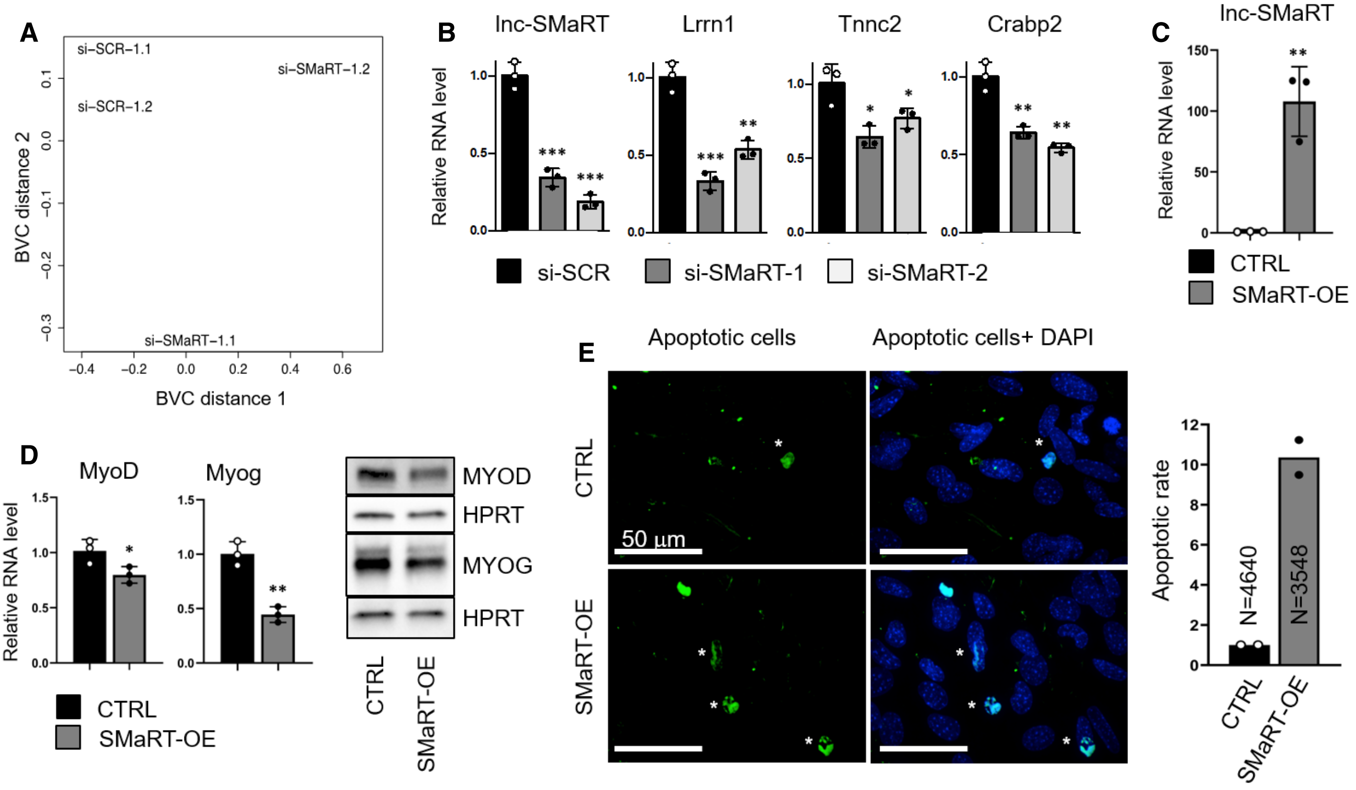

Figure EV2. Lnc-SMaRT depletion and overexpression in C2C12 cells.

A Multi-dimensional scaling plot (MDS) of the RNA-Seq data (si-SCR and si-SMaRT treated C2C12 samples) in which distances were derived from leading biological coefficient of variation (BCV) between each pair of RNA samples.

B qPCR analysis of indicated deregulated mRNAs identified by RNA-seq data in C2C12 cells undergoing differentiation (D2) in control samples (black bars) or samples depleted for lnc-SMaRT with two different siRNAs (gray and white bars). The RNA expression levels in qPCR analyses were normalized against GAPDH mRNA and expressed as relative quantities with respect to the si-SCR sample set to a value of 1. Data are presented as the mean ± s.e.m. of three biological replicates (dots). Statistical analysis was performed with ordinary analysis of variance (ANOVA) followed by Dunnett's multiple comparison test. *$P < 0.05$, **$P < 0.01$, ***$P < 0.001$.

C qPCR analysis of lncSMaRT expression in C2C12 stable cell lines undergoing differentiation (D1.5) in control samples (black bars) or samples overexpressing lnc-SMaRT (gray bars). The RNA expression levels in qPCR analyses were normalized against GAPDH mRNA and expressed as relative quantities with respect to the control sample (CTRL) set to a value of 1. Data are presented as the mean ± s.e.m of three biological replicates (dots). Statistical analysis was performed with unpaired two-tailed *t*-test. **$P < 0.01$.

D Left panel: qPCR analysis of MyoD and Myog RNA expression in C2C12 stable cell lines undergoing differentiation (D1.5) in control samples (black bars) or samples overexpressing lnc-SMaRT (gray bars). The RNA expression levels in qPCR analyses were normalized against GAPDH mRNA and expressed as relative quantities with respect to the control sample (CTRL) set to a value of 1. Data are presented as the mean ± s.e.m of three biological replicates (dots). Statistical analysis was performed with unpaired two-tailed *t*-test. *$P < 0.05$, **$P < 0.01$. Right panel: Western blot on protein extract from control and lncSMaRT-overexpressing C2C12 stable cell lines undergoing differentiation (D1.5). MYOD and MYOG antibodies were used. HPRT was used as loading control. Representative results from three independent experiments are shown.

E Detection of apoptosis by TUNEL assay in C2C12 cell line. CTRL: control C2C12 cell line transfected with the empty vector; lnc-SMaRT: C2C12 cell line overexpressing lnc-SMaRT. Histogram represents the mean of apoptotic rate percentage (apoptotic cells/total nuclei*100) from two independent biological samples (dots); the total number of analyzed cells is indicated in the bars.

Source data are available online for this figure.

Figure EV3. Effects of SMaRT and DHX36 depletion on Mlx mRNA levels.

A qPCR on RNAs obtained from psoralen-crosslinking lnc-SMaRT pull-down in C2C12 cells two days after the switch to differentiation. Left panel: lnc-SMaRT pull-down performed in different crosslinking conditions (+Psoralen +UV, +Psoralen −UV, -Psoralen +UV). Right panel: lnc-SMaRT pull-down performed in C2C12 cells treated with a control siRNA (si-SCR) or siRNA against lnc-SMaRT (si-SMaRT-1). Two sets of probes were used (Probe Set #1 and Probe Set #2); a set of probes against LacZ mRNA (LacZ) was used as a negative control. Values are expressed as fold-change of percentage of input over LacZ background. GAPDH was used as negative control. Data are presented as the mean ± s.e.m of three biological replicates (dots). Statistical analysis was performed with two-way ANOVA followed by Sidak's multiple comparisons test.

B Schematic representation of the three MLX isoforms (in blue, CDS: thick lines, UTR: thin lines). The position of the two interacting regions on lnc-SMaRT is highlighted with red boxes, together with the predicted pairing sequences.

C Sequences of complementarity between lncSMaRT and Mlx.

D RT–PCR analysis of lnc-SMaRT, Dhx36, Mlx-α, Mlx-β, and Mlx-γ expression on RNA extract from C2C12 cells treated with siRNAs against lnc-SMaRT (si-SMaRT-1, si-SMaRT-2), DHX36 (si-DHX36), or a control siRNA (si-SCR) collected 2 days after switching to differentiation medium. GAPDH was used as a control. Representative results from at least three independent experiments are shown.

E qPCR analysis of DHX36 expression in N2a cells transfected with an empty vector (CTRL) or with a plasmid overexpressing flagged MLX-γ (MLX-γ) in control conditions (si-SCR) or in knockdown of DHX36 (si-DHX36). The RNA expression levels in qPCR analyses were normalized against GAPDH mRNA and expressed as relative quantities with respect to the control sample (CTRL) set to a value of 1. Data are presented as the mean ± s.e.m of three biological replicates (dots). Statistical analysis was performed with unpaired two-tailed *t*-test. ***$P < 0.001$.

Source data are available online for this figure.

 © 2020 The Authors

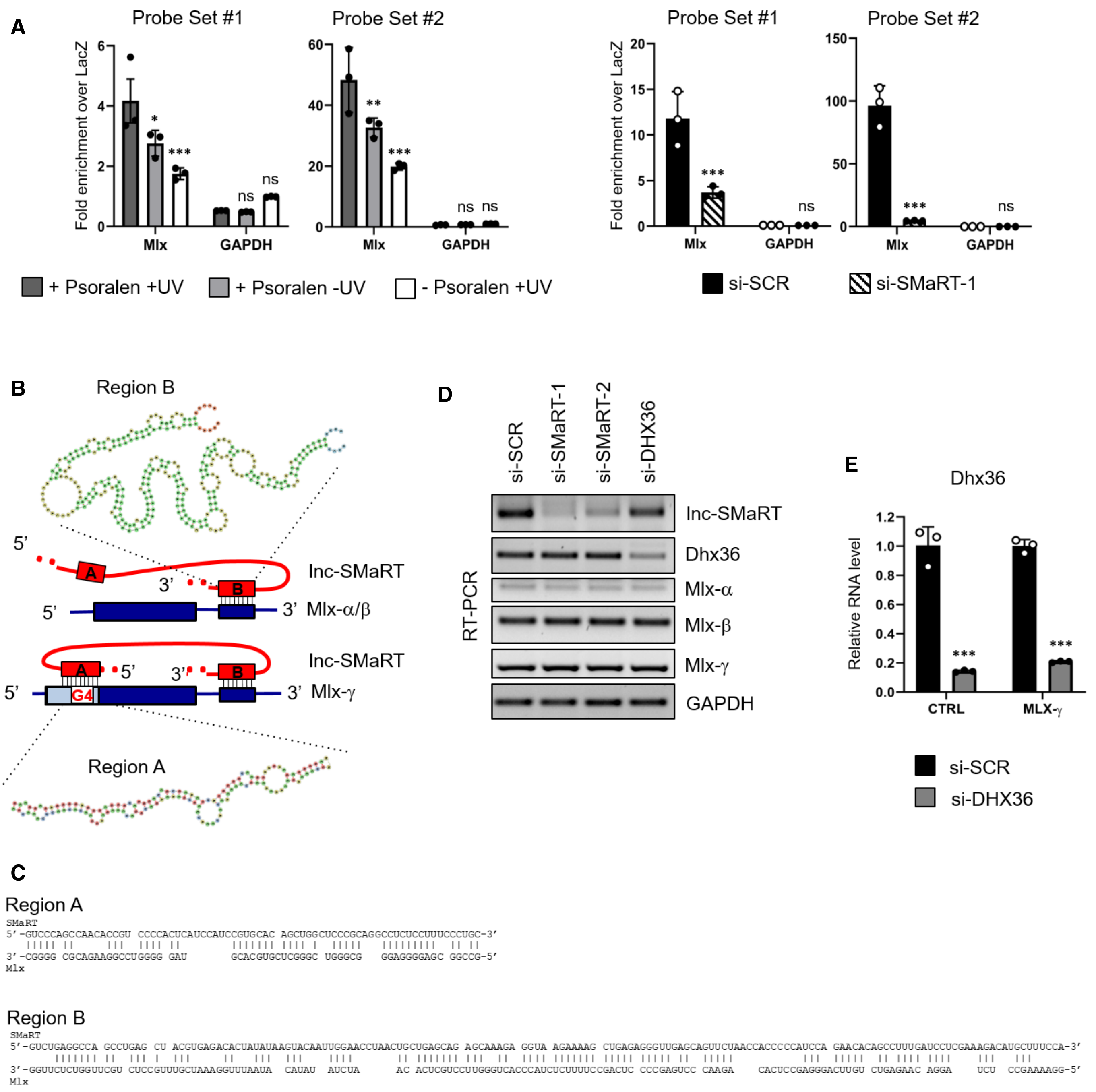
A
Probe Set #1
Probe Set #2
Fold enrichment over LacZ
Mlx
GAPDH
ns
+ Psoralen +UV
+ Psoralen -UV
- Psoralen +UV
si-SCR
si-SMaRT-1
B
Region B
5'
3'
A
B
lnc-SMaRT
Mlx-α/β
Mlx-γ
G4
Region A
D
si-SCR
si-SMaRT-1
si-SMaRT-2
si-DHX36
RT-PCR
lnc-SMaRT
Dhx36
Mlx-α
Mlx-β
Mlx-γ
GAPDH
E
Dhx36
Relative RNA level
CTRL
MLX-γ
si-SCR
si-DHX36
C
Region A
SMaRT
5'-GUCCCAGCCAACACCGU CCCCACUCAUCCAUCCGUGCAC AGCUGGCUCCCGCAGGCCUCUCCUUUCCCUGC-3'
3'-CGGGG CGCAGAAGGCCUGGGG GAU GCACGUGCUCGGGC UGGGCG GGAGGGGAGC GGCCG-5'
Mlx
Region B
SMaRT
5'-GUCUGAGGCCA GCCUGAG CU ACGUGAGACACUAUAUAAGUACAAUUGGAACCUAACUGCUGAGCAG AGCAAAGA GGUA AGAAAAG CUGAGAGGGUUGAGCAGUUCUAACCACCCCCAUCCA GAACACAGCCUUUGAUCCUCGAAAGACAUGCUUUCCA-3'
3'-GGUUCUCUGGUUCGU CUCCGUUUGCUAAAGGUUUAAUA CAUAU AUCUA AC ACUCGUCCUUGGGUCACCCAUCUCUUUUCCGACUC CCCGAGUCC CAAGA CACUCCGAGGGACUUGU CUGAGAAC AGGA UCU CCGAAAAGG-5'
Mlx

Figure EV3.

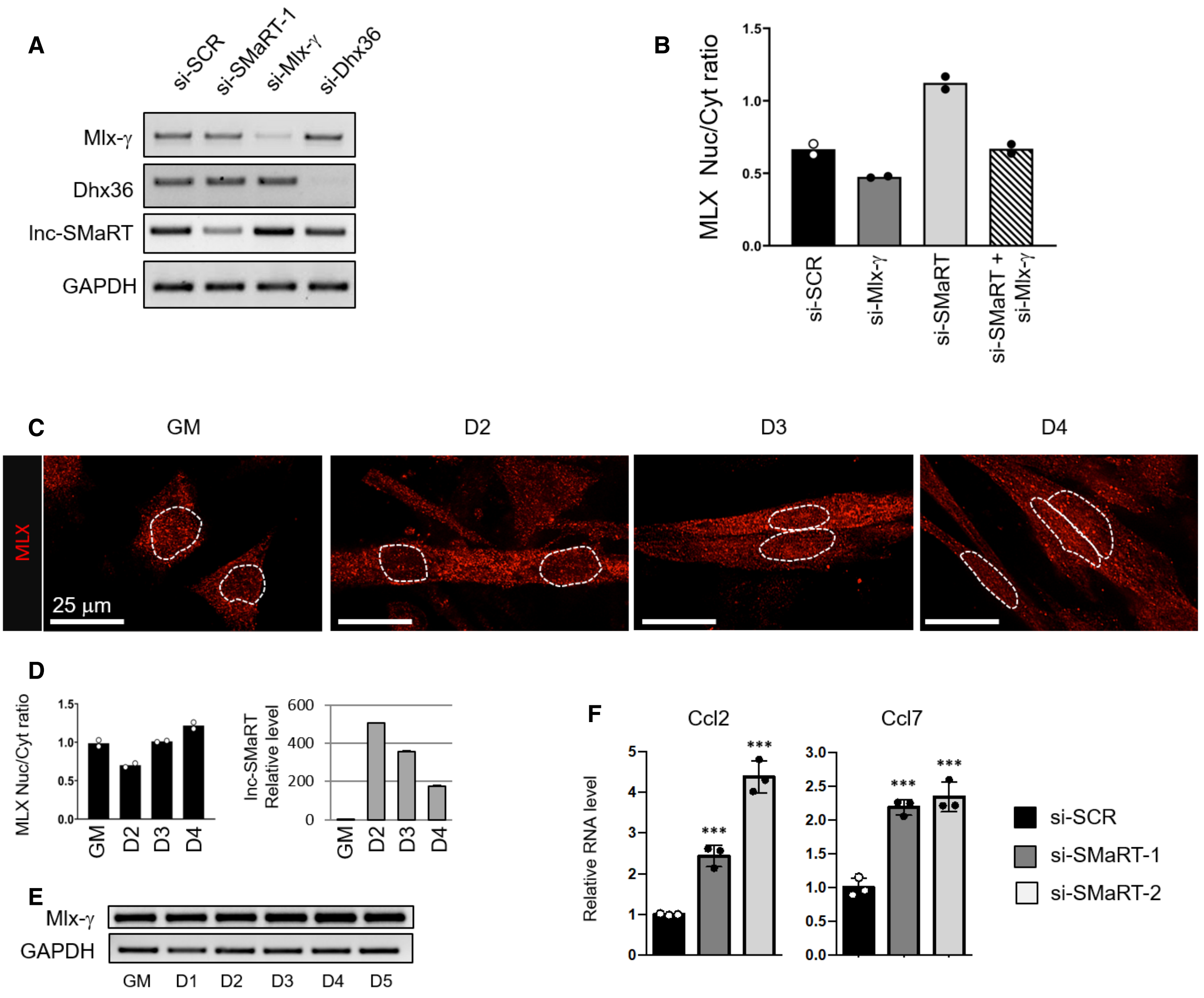

Figure EV4. MLX subcellular localization is modulated during C2C12 differentiation.

A RT–PCR analysis of Mlx-γ, Dhx36, and lnc-SMaRT expression on RNA extracted from C2C12 cells treated with siRNAs against Mlx-γ (si-Mlx-γ), Dhx36 (si-Dhx36), lnc-SMaRT (si-SMaRT-1), or a control siRNA (si-SCR) collected 2 days after switching to differentiation medium. GAPDH was used as a control. Representative results from at least three independent experiments are shown.

B Histogram representing the fluorescence intensity ratio of the MLX protein signals in the nuclear (Nuc) and cytoplasmic (Cyt) compartments in the indicated conditions. About 20 cells from two independent experiments were analyzed as indicated in Materials and Methods. Data are presented as the mean of two biological replicates (dots).

C Representative images of MLX immunofluorescence (red signal) performed on C2C12 cells undergoing differentiation at the indicated time points. Dashed lines indicate the edge of the nucleus.

D Left panel: Histogram representing the ratio between the mean intensity of MLX protein signal in the nuclear and cytoplasmic compartments in C2C12 cells undergoing differentiation at the indicated time points. About 50 cells from two independent experiments were analyzed as indicated in Materials and Methods. Data are presented as the mean of two biological replicates (dots). Right panel: qPCR analysis of lnc-SMaRT RNA expression in C2C12 cells undergoing differentiation at the indicated time points. The RNA expression levels in qPCR analyses were normalized against GAPDH mRNA and expressed as relative quantities with respect to GM samples set to a value of 1.

E RT–PCR analysis of Mlx-γ mRNA expression in C2C12 cells undergoing differentiation at the indicated time points. GAPDH was used as a control. Representative results from at least three independent experiments are shown.

F qPCR analysis of indicated mRNAs in C2C12 cells undergoing differentiation (D2) in control samples (black bars) or samples depleted for lnc-SMaRT with two different siRNAs (gray and white bars). The RNA expression levels in qPCR analyses were normalized against GAPDH mRNA and expressed as relative quantities with respect to the si-SCR sample set to a value of 1. Data are presented as the mean ± s.e.m. of three biological replicates (dots). Statistical analysis was performed with ordinary analysis of variance (ANOVA) followed by Dunnett's multiple comparison test. ***$P < 0.001$.

Source data are available online for this figure.

 © 2020 The Authors

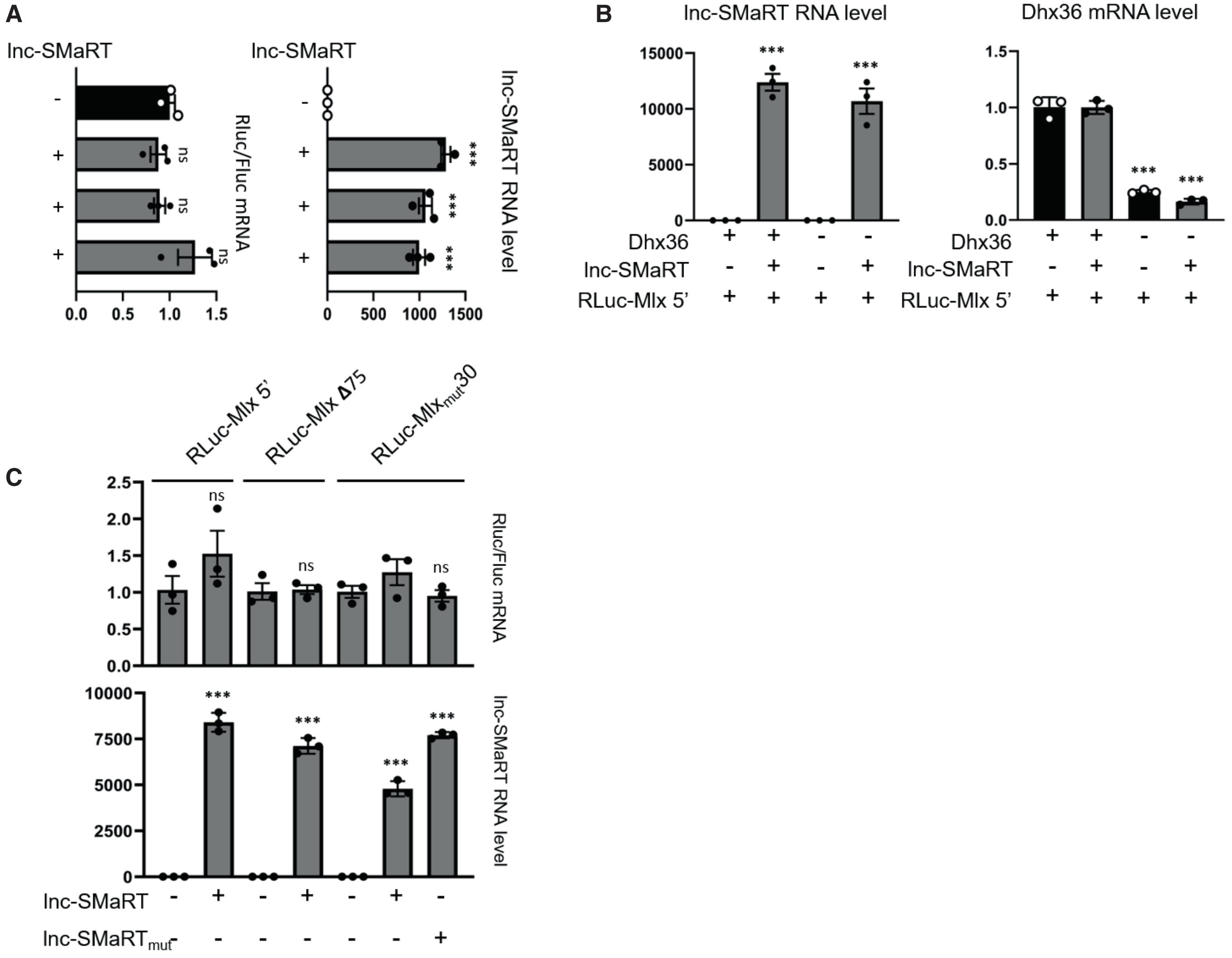

Figure EV5. Lnc-SMaRT controls the translation of Mlx-γ.

A Left panel: qPCR analysis of luciferase mRNAs expression on the same samples analyzed in Fig 5A. The RLuc RNA expression levels in qPCR analyses were normalized against FLuc RNA and expressed as relative quantity with respect to lnc-SMaRT (–) sample set to a value of 1. Right panel: qPCR analysis of lnc-SMaRT RNA expression on the same samples analyzed in Fig 5A. The RNA expression levels in qPCR analyses were normalized against GAPDH mRNA and expressed as relative quantities with respect to the lnc-SMaRT (–) sample set to a value of 1. Data are presented as the mean ± s.e.m. of three biological replicates (dots). Statistical analysis was performed with ordinary analysis of variance (ANOVA) followed by Dunnett's multiple comparison test. ***$P < 0.001$.

B Left panel: qPCR analysis of lnc-SMaRT RNA expression on the same samples analyzed in Fig 5B. The RNA expression levels in qPCR analyses were normalized against GAPDH mRNA and expressed as relative quantities with respect to the lnc-SMaRT (–) sample set to a value of 1. Right panel: qPCR analysis of Dhx36 RNA expression on the same samples analyzed in Fig 5B. The RNA expression levels in qPCR analyses were normalized against GAPDH mRNA and expressed as relative quantities with respect to the control siRNA treated sample set to a value of 1. Data are presented as the mean ± s.e.m. Statistical analysis was performed with two-way ANOVA followed by Sidak's multiple comparisons test. ***$P < 0.001$.

C Upper panel: qPCR analysis of luciferase mRNAs expression on the same samples analyzed in Fig 5C. The RLuc RNA expression levels in qPCR analyses were normalized against FLuc RNA and expressed as relative quantity with respect to lnc-SMaRT (–) sample set to a value of 1. Lower panel: qPCR analysis of lnc-SMaRT RNA expression on the same samples analyzed in Fig 5C. The RNA expression levels in qPCR analyses were normalized against GAPDH mRNA and expressed as relative quantities with respect to the lnc-SMaRT (–) sample set to a value of 1. Data are presented as the mean ± s.e.m. of three biological replicates (dots). Statistical analysis was performed with ordinary analysis of variance (ANOVA) followed by Tukey's multiple comparison test. ***$P < 0.001$.

Source data are available online for this figure.

© 2020 The Authors
