# Supplementary material for: SMaRT lncRNA controls translation of a G‐quadruplex‐containing mRNA antagonizing the DHX36 helicase
Source: EMBO Rep. 2020 Apr 26;21(6):e49942. doi: 10.15252/embr.201949942 (PMC7271651; doi:10.15252/embr.201949942)
Supplement: Supplementary file 3 — Table EV2 [file EMBR-21-e49942-s003.docx]

**Table EV2**. **Read numbers and mapping statistics related to the lnc-SMaRT depletion RNA-Seq experiment.**

| **Samples** | **Raw read pairs** | **Read pairs after pre-processing** | **Read pairs after rRNAs and tRNAs filtering** | **Mapped read pairs** | **Percentage of multiple alignments** |
| --- | --- | --- | --- | --- | --- |
| siSCR_1.1 | 20035463 | 19777347 | 19443783 | 18339541 | 13.90% |
| siSCR_1.2 | 21719136 | 21434097 | 21054764 | 19803695 | 11.90% |
| si-SMaRT_1.1 | 23346288 | 23043533 | 16344544 | 15158622 | 10.00% |
| si-SMaRT_1.2 | 21418462 | 20974553 | 20888806 | 19949803 | 7.90% |
