# Supplementary material for: SMaRT lncRNA controls translation of a G‐quadruplex‐containing mRNA antagonizing the DHX36 helicase
Source: EMBO Rep. 2020 Apr 26;21(6):e49942. doi: 10.15252/embr.201949942 (PMC7271651; doi:10.15252/embr.201949942)
Supplement: Supplementary file 4 — Table EV3 [file EMBR-21-e49942-s004.docx]

**Table EV3**. **Read numbers and mapping statistics related to the lnc-SMaRT pull-down experiment.**

| **Samples** | **Raw reads** | **Reads after pre-processing** | **Reads after rRNAs and tRNAs filtering** | **Mapped reads** | **Percentage of multiple alignments** | **Mapped reads after mithocondrial RNA filtering** | **Reads after duplicate filtering** | **Reads after multi-mapped filtering** |
| --- | --- | --- | --- | --- | --- | --- | --- | --- |
| lnc-SMaRT Pull-Down | 70312265 | 68617565 | 66753421 | 61946867 | 8.8% | 59286790 | 5777943 | 773686 |
| LacZ | 68544492 | 67152108 | 66064361 | 54286366 | 15.8% | 52276994 | 9256961 | 930750 |
| Input | 28684619 | 28511611 | 27841521 | 27430205 | 15.2% | 26004129 | 10047635 | 6186390 |
