# Supplementary material for: SMaRT lncRNA controls translation of a G‐quadruplex‐containing mRNA antagonizing the DHX36 helicase
Source: EMBO Rep. 2020 Apr 26;21(6):e49942. doi: 10.15252/embr.201949942 (PMC7271651; doi:10.15252/embr.201949942)
Supplement: Supplementary file 5 — Table EV4 [file EMBR-21-e49942-s005.docx]

**Table EV4. List of primers, biotinylated probes and siRNA sequences.**

| **Primer Name** | **Primer Sequence** |
| --- | --- |
| lnc-SMaRT Fw | CTAGCAGCAGCACTCACAGC |
| lnc-SMaRT Rv | CTTGTGCCTCGTTGACAAAA |
| pre-GAPDH Fw | GTATGTATGGGGAGAGCTGG |
| GAPDH Fw | TGACGTGCCGCCTGGAGAAA |
| GAPDH Rv | AGTGTAGCCCAAGATGCCCTTCAG |
| MYOD Fw | GCAGAATGGCTACGACACC |
| MYOD Rv | CACTATGCTGGACAGGCAGT |
| MYOG Fw | TCCCAACCCAGGAGATCATT |
| MYOG Rv | CATATCCTCCACCGTGATGC |
| MEF2C Fw | ACGAGGATAATGGATGAGCGT |
| MEF2C Rv | TCACAGTCGCACAGCACG |
| MCK Fw | TTACACTCTGCCTCCGCACT |
| MCK Rv | GTACTTGCCCTTGAACTCGC |
| Dys Fw | AGCTCAACCGTCGATTTGCAGC |
| Dys Rv | TTCAGCCTCCAGTGGTTCAAGC |
| LRRN1 Fw | GAAGATCGACAACCCCCACA |
| LRRN1 Rv | GGACACCGTGAGACACACTT |
| TNNC2 Fw | AGCGAAGAGGAACTGGCTGAGT |
| TNNC2 Rv | CGATCTCCTCTTCTGTCACATGC |
| CRABP2 Fw | GTGGATGGGAGACCCTGTAAG |
| CRABP2 Rv | TCATTGGTCAGTTCTCGGCTC |
| MLX ex4 Fw | GGAGTCCTACAAAGACCGGA |
| MLX ex5 Rv | GGCAGGTAGGGACAATGGT |
| MLX ex2 Fw | TACAGTGACACCAGCCTG |
| MLX total ex1 Fw | CTGGCTTGTTTCCGGTTCGG |
| MLX ex3 Rv | CTCTGGACACTACACTCCCC |
| MLXγ ex1 Fw | GCAGAGAAGACAGCTCTCACC |
| MLX ex2 Rv | GCTGTTGTCACTGTAGGCAT |
| DHX36 Fw | GCTGAGCATCTTCTTGGAGC |
| DHX36 Rv | AAACCAGCACAGATGACAGC |
| CCL2 Fw | CTCACCTGCTGCTACTCATTCAG |
| CCL2 Rv | GTCAGCACAGACCTCTCTCTTG |
| CCL7 Fw | CATCCACATGCTGCTATGTC |
| CCL7 Rv | GCAGACTTCCATGCCCTTCT |
| Firefly LUC Fw | TGCAGAAGATCCTGAACGTG |
| Firefly LUC Rv | CGGTAGACCCAGAGCTGTTC |
| Renilla LUC Fw | TCGTCCATGCTGAGAGTGTC |
| Renilla LUC Rv | CTAACCTCGCCCTTCTCCTT |
| 3'UTR Mlx XhoI Fw | AAAACTCGAGGCAGAGCAGCCAACAAGAG |
| 3'UTR Mlx NotI Rv | AAAAGCGGCCGCCTGGGGAAGGCAGTAGGAA |
| ΨCHECK2 ΔATG Fw | GCTTCCAAGGTGTACGACCC |
| ΨCHECK2 ΔATG Rv | GGTGGCTAGCCTATAGTGAGTC |
| Rluc-Mlx 5' Fw | TATAGGCTAGCCACCTGACTGAGCCTCGCCTCTTCCT |
| Rluc-Mlx 5' Rv | GTACACCTTGGAAGCGTTGTCACTGTAGGCATACTCGAC |
| Rluc-Mlx Δ75 Fw | TCCCCAACTATCCCCAGAGTC |
| Rluc-Mlx Δ75 Rv | GGGAGACTCTGGGGATAGTT |
| Rluc-Mlx mut30 Fw | CCGCAGGCCTCTCCTTTAGGGGGTCCGGAAGACG |
| Rluc-Mlx mut30 Rv | GAGCCAGCTGTGCACGGCCGGCGTGCGCGTC |
| lnc-SMaRT XhoI Fw | AAAACTCGAGACTAGTCACTGGAGCTAGC |
| lnc-SMaRT NotI Rv | AAAAGCGGCCGCGGGCATGGTATAACAACGT |
| lnc-SMaRT mut Fw | CGGGCTCGTGCACGTCCCTGCATAGCCAATTATTCC |
| lnc-SMaRT mut Rv | ACCCGCCCTCCCCTCGATGGATGAGTGGGGA |
| lnc-SMaRT stable line Fw | AGCACAGTGGCGGCCGGGCATGGTATACAACGT |
| lnc-SMaRT stable line Rv | TTGGAACCTAACTGCGCACTTTACCCACTGAGC |

| **Biotinylated Oligonucleotides**  **used for lnc-SMaRT pulldown** | **Sequence** |
| --- | --- |
| Set #1, probe 1 | TAGCTAGCTCCAGTGACTAG |
| Set #1, probe 2 | AACTAGAACCCCCAAACAGA |
| Set #1, probe 3 | CAGCAGTTAGGTTCCAATTG |
| Set #1, probe 4 | TGGGTAAAGTGCTTGATGCA |
| Set #1, probe 5 | ACACGGGCATGGTATACAAC |
| Set #1, probe 6 | TGTGCCTTCATGTGGGGAAG |
| Set #2, probe 7 | TAGGAAGCAAGACCGTCATC |
| Set #2, probe 8 | GTCTTTCGAGGATCAAAGGC |
| Set #2, probe 9 | TACTGCTCTCATCATTTTGC |
| LacZ, probe 1 | AATGTGAGCGAGTAACAACC |
| LacZ, probe 2 | ATTAAGTTGGGTAACGCCAG |
| LacZ, probe 3 | AATAATTCGCGTCTGGCCTT |
| LacZ, probe 4 | AATTCAGACGGCAAACGCT |
| LacZ, probe 5 | ATCTTCCAGATAACTGCCGT |

| **siRNAs** | **Sequence** |
| --- | --- |
| si-SMaRT-1 | GAGAAGCAGAACAAACAGAUA |
| si-SMaRT-2 | CAGAAACUCUCUGCUUUCGUA |
| si-Mlx-γ | CCAACUAUCCCCAGAGUCUUU |
