# Supplementary figures and images for: SMaRT lncRNA controls translation of a G‐quadruplex‐containing mRNA antagonizing the DHX36 helicase
Source: EMBO Rep. 2020 Apr 26;21(6):e49942. doi: 10.15252/embr.201949942 (PMC7271651; doi:10.15252/embr.201949942)

Uncropped Western Blot files Figures EV1

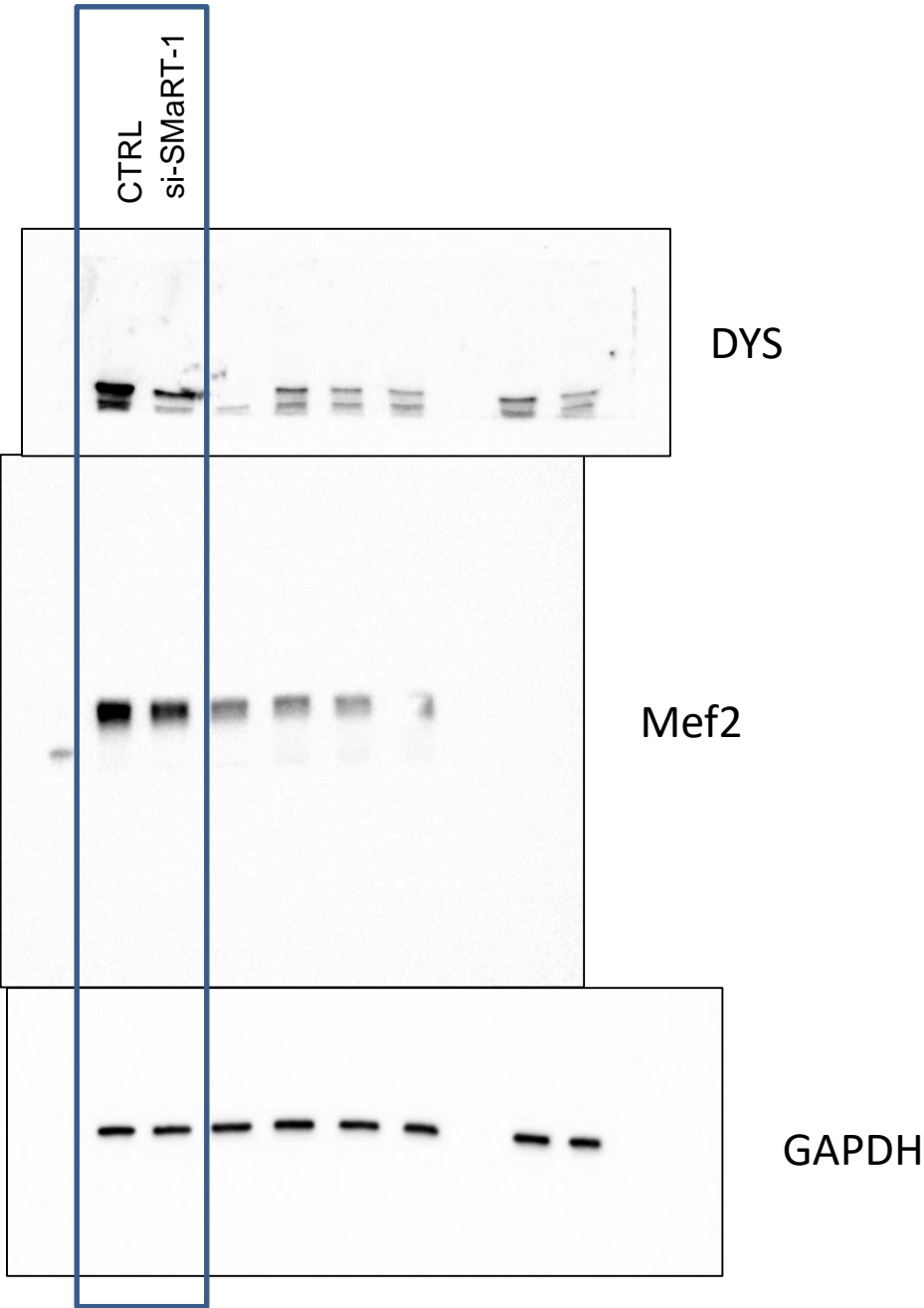

FIGURE EV1D

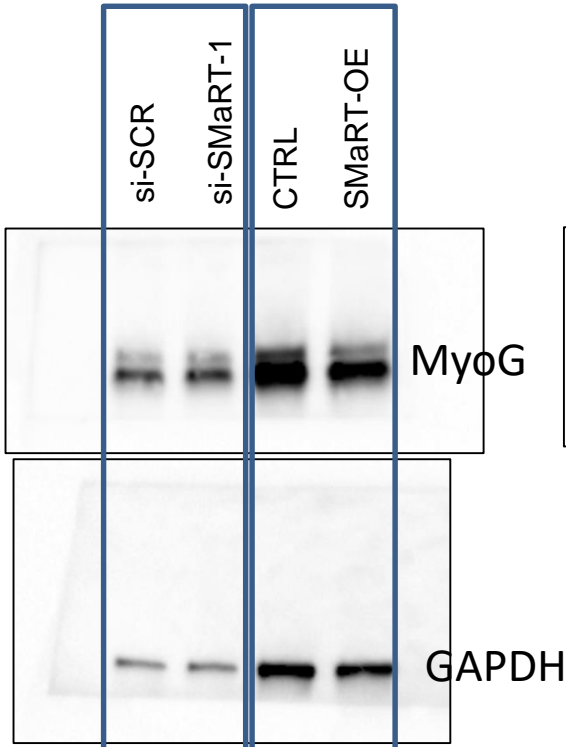

FIGURE EV1D

FIGURE EV1H

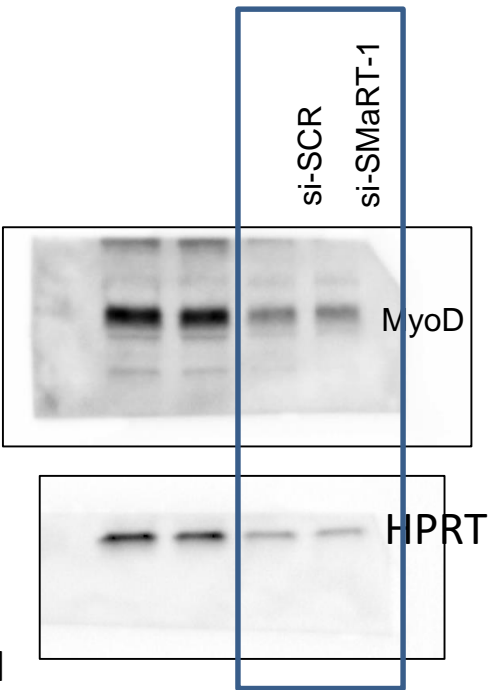

FIGURE EV1D

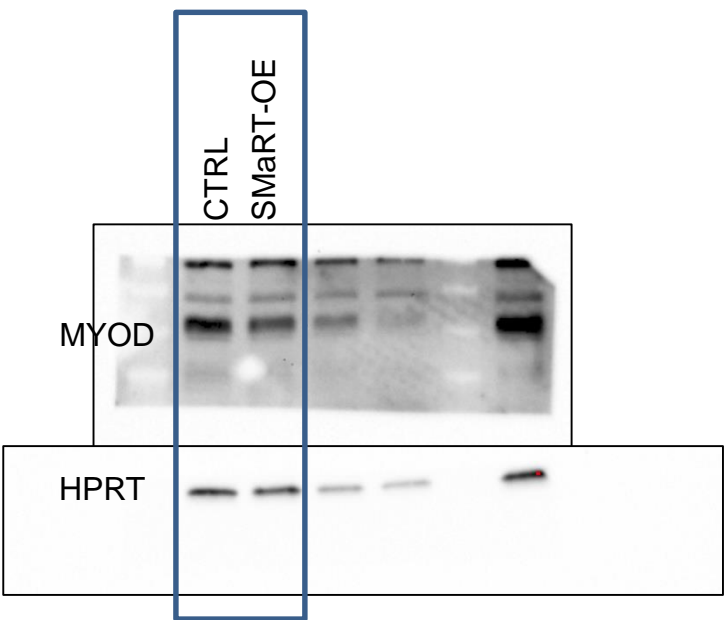

FIGURE EV1H

Supplement: Supplementary file 9 — Source Data for Expanded View [file EMBR-21-e49942-s013.zip › 49942_EV_Figure_source_data/FigEV1_source_data/FigEV1_source_data.pdf]

Uncropped Western Blot files Figure 2

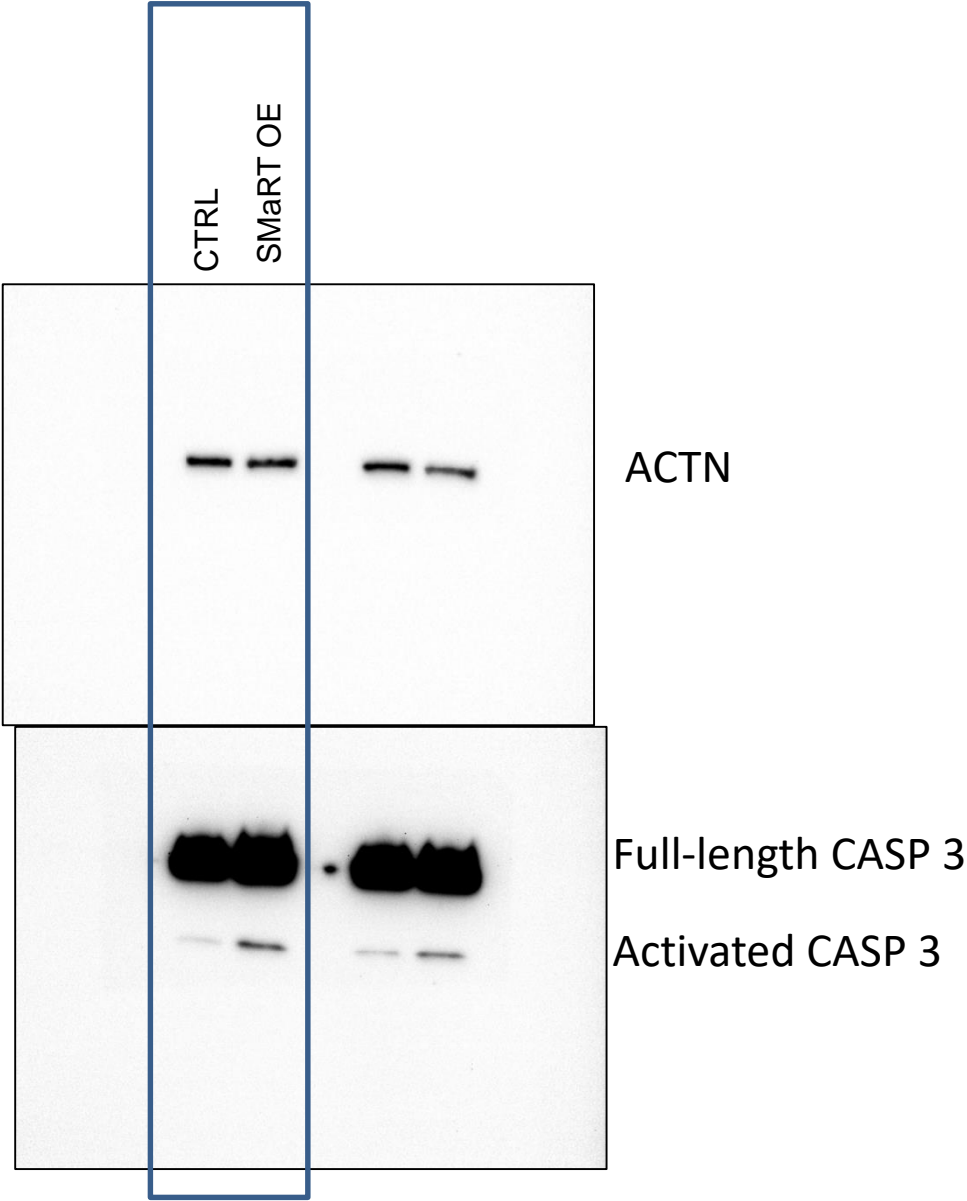

FIGURE 2D

Supplement: Supplementary file 12 — Source Data for Figure 2 [file EMBR-21-e49942-s010.pdf]
