# Supplementary material for: SMaRT lncRNA controls translation of a G‐quadruplex‐containing mRNA antagonizing the DHX36 helicase
Source: EMBO Rep. 2020 Apr 26;21(6):e49942. doi: 10.15252/embr.201949942 (PMC7271651; doi:10.15252/embr.201949942)

FIGURE 3B

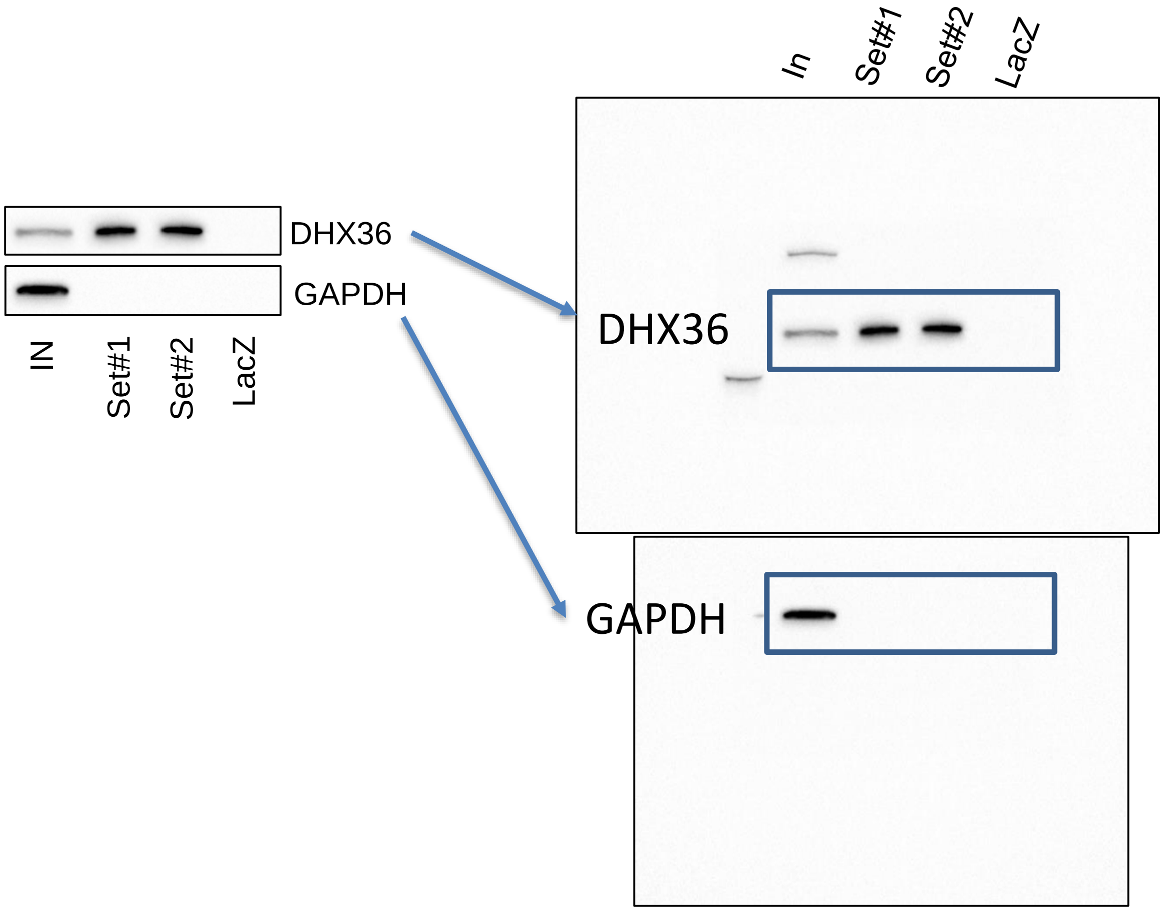

FIGURE 3C

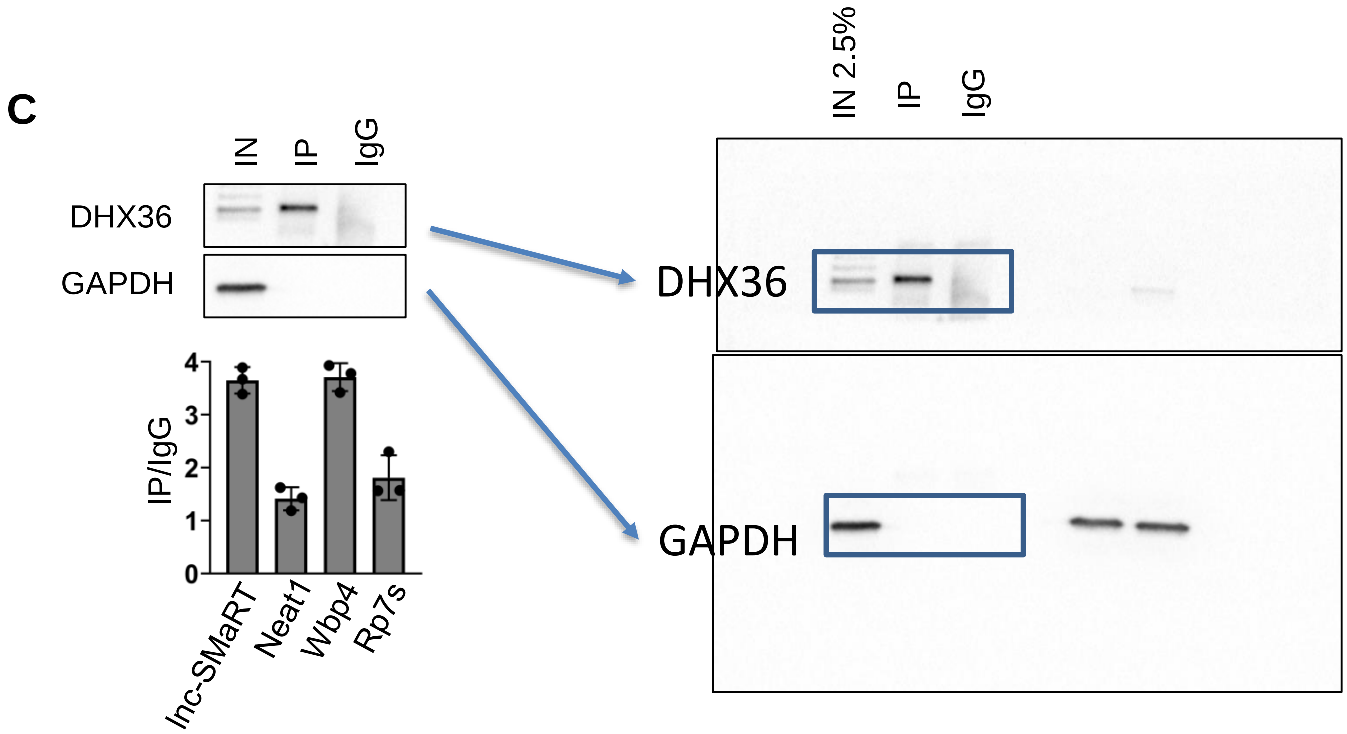

FIGURE 3C

FIGURE 3D

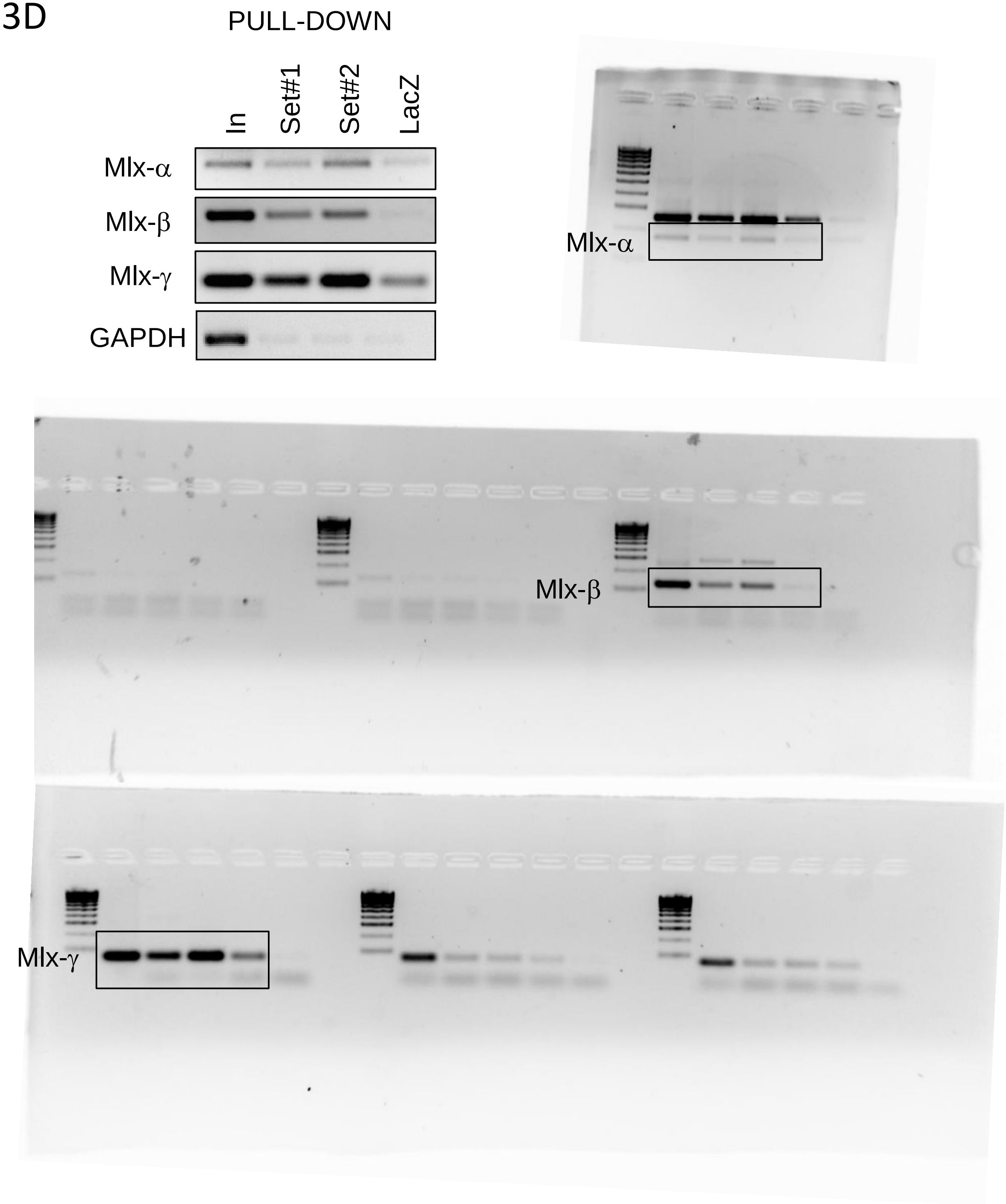
PULL-DOWN
In
Set#1
Set#2
LacZ
Mlx-α
Mlx-β
Mlx-γ
GAPDH
Mlx-α
Mlx-β
Mlx-γ

FIGURE 3E

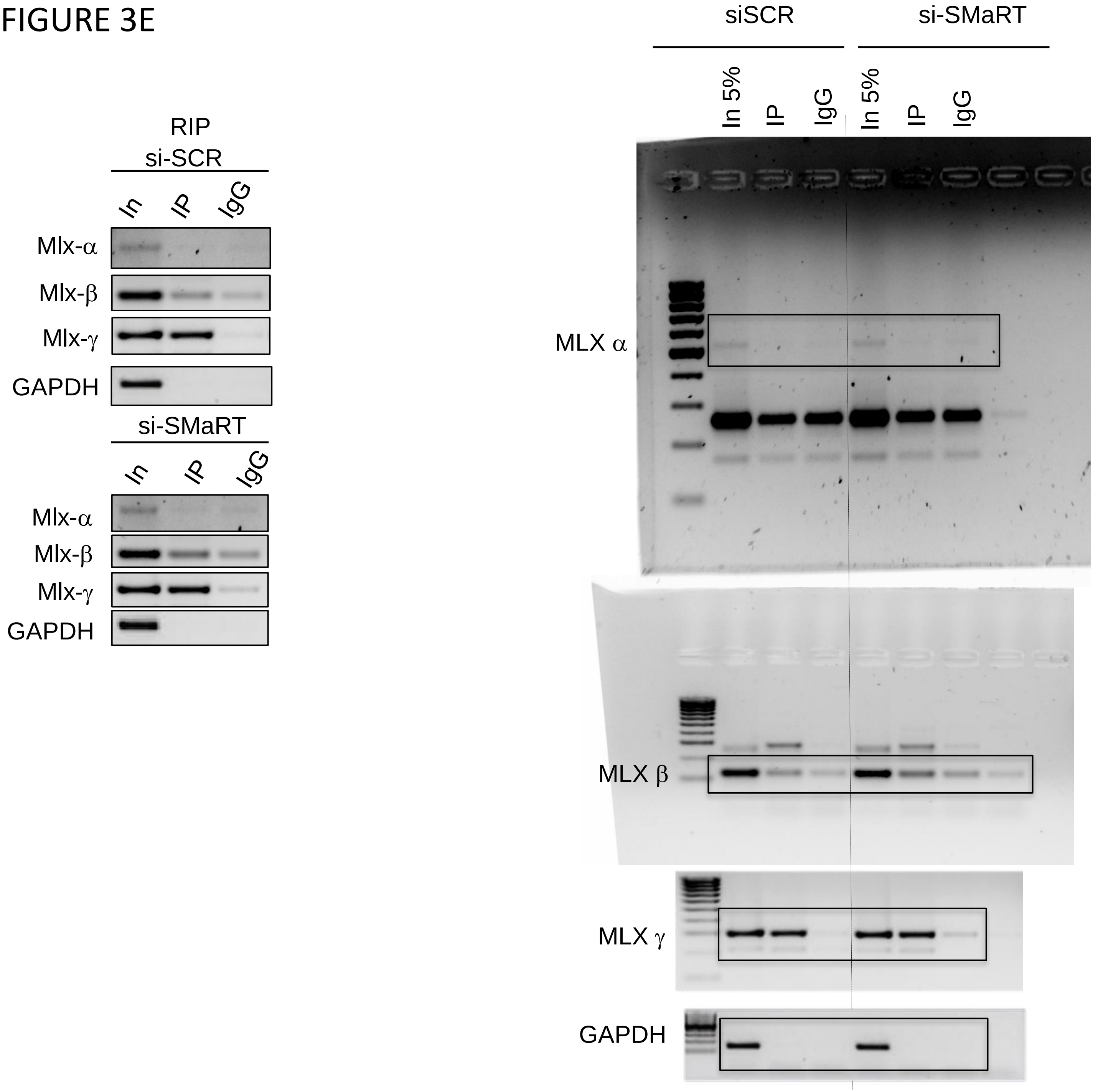
RIP
si-SCR
In
IP
IgG
Mlx-α
Mlx-β
Mlx-γ
GAPDH
si-SMaRT
In
IP
IgG
Mlx-α
Mlx-β
Mlx-γ
GAPDH
siSCR
si-SMaRT
In 5%
IP
IgG
In 5%
IP
IgG
MLX α
MLX β
MLX γ
GAPDH

FIGURE 3F WESTERN BLOT

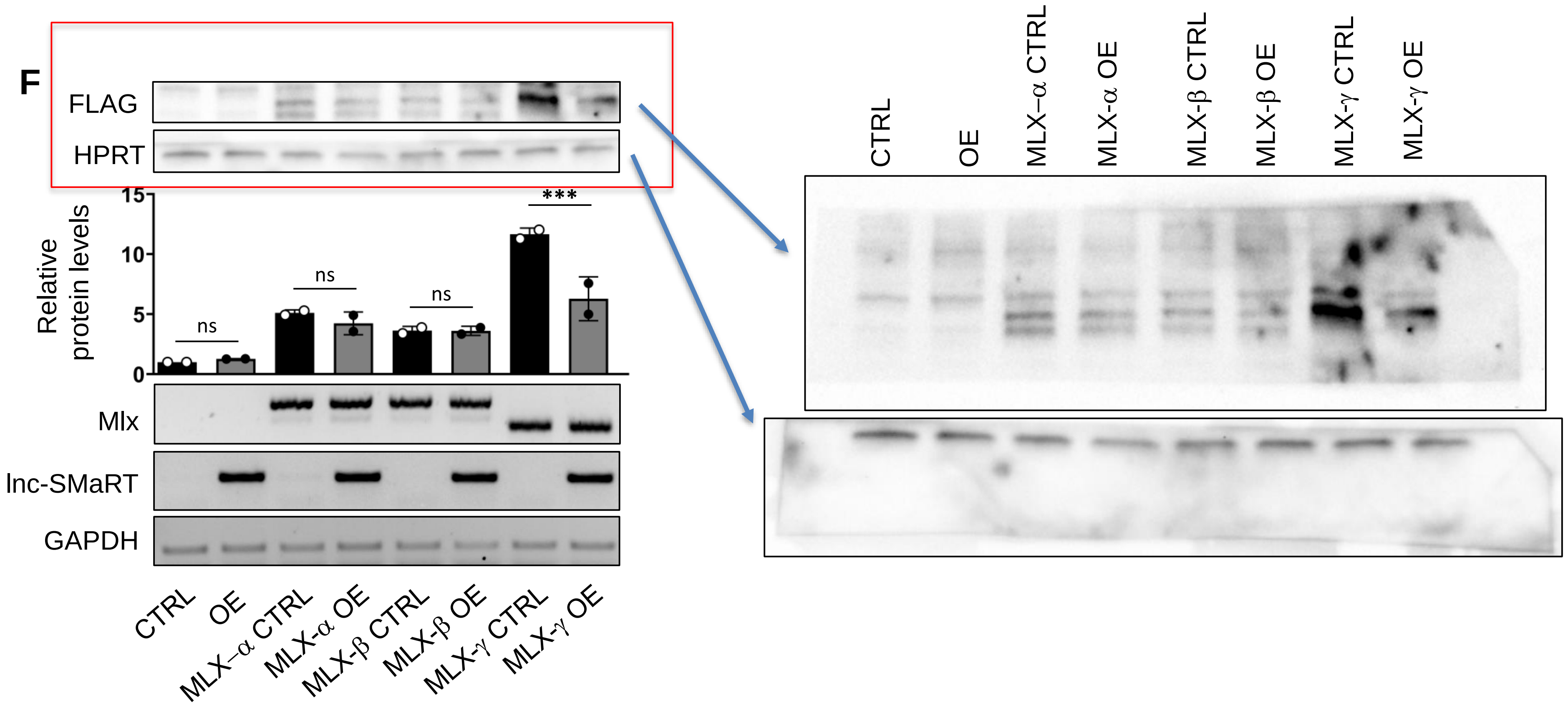

FIGURE 3F RT-PCR gels

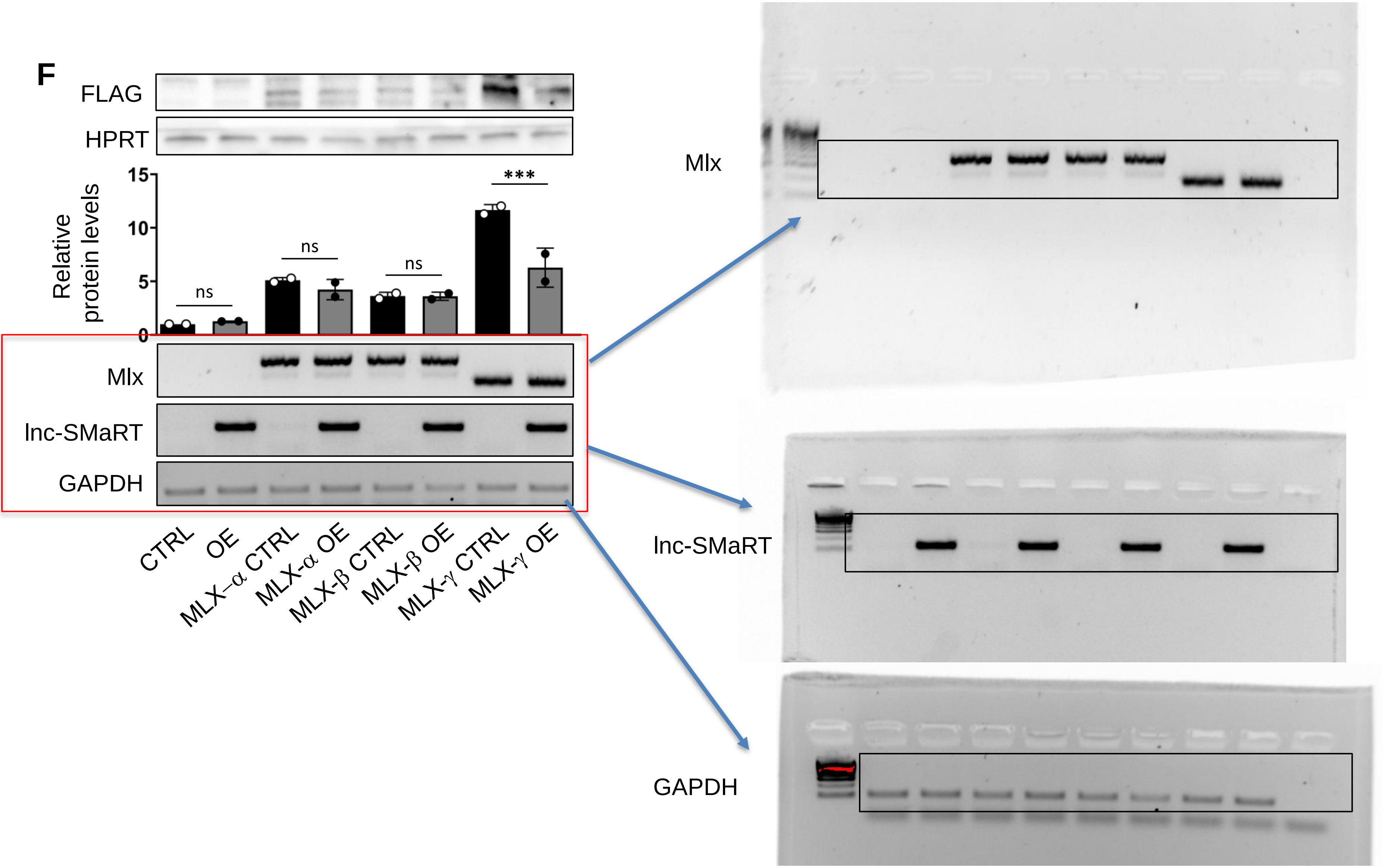

FIGURE 3G WESTERN BLOT

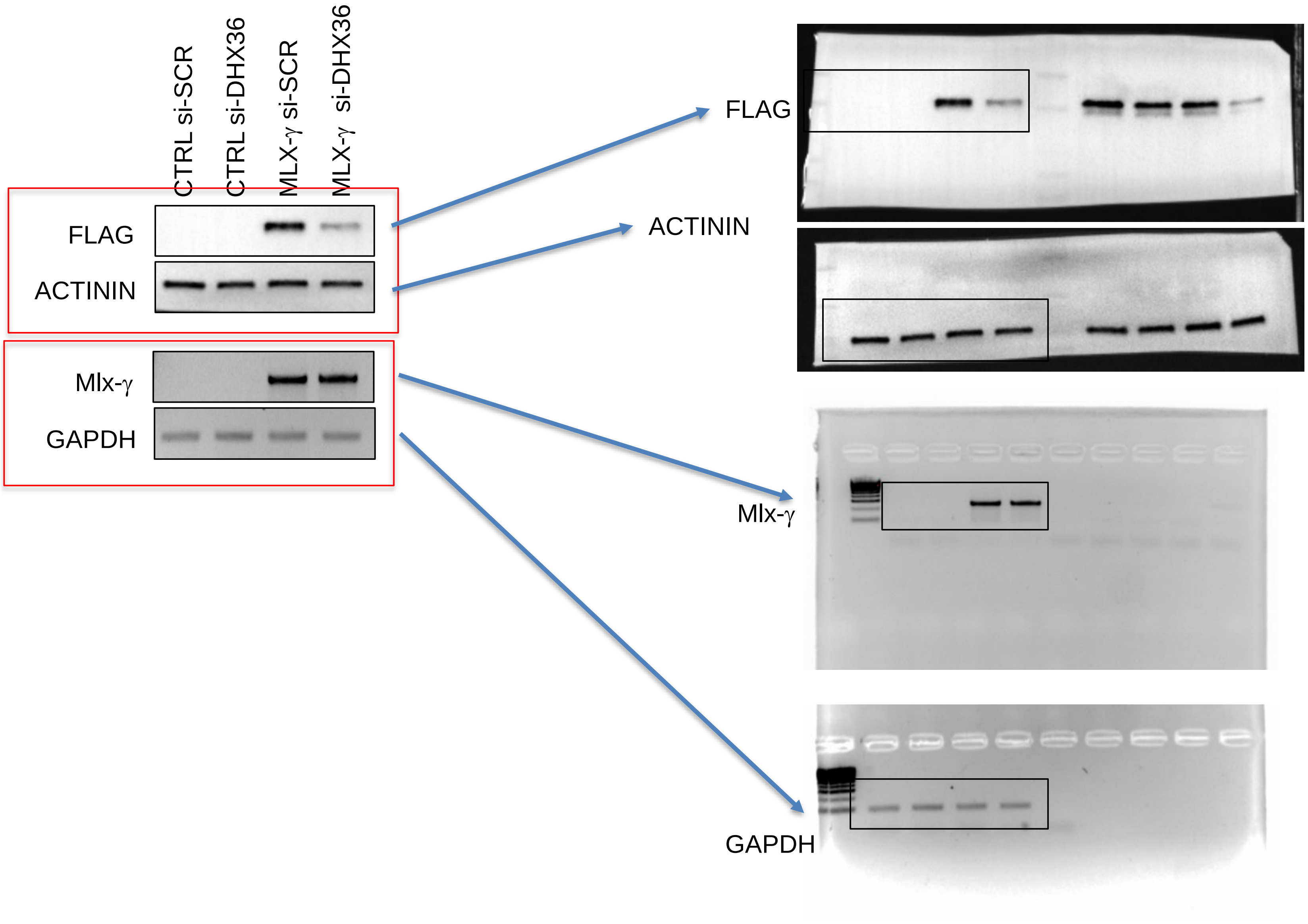

Supplement: Supplementary file 13 — Source Data for Figure 3 [file EMBR-21-e49942-s011.zip › 49942_Fig3_source_data/fig_3_source_data.pdf]
