# Supplementary material for: SMaRT lncRNA controls translation of a G‐quadruplex‐containing mRNA antagonizing the DHX36 helicase
Source: EMBO Rep. 2020 Apr 26;21(6):e49942. doi: 10.15252/embr.201949942 (PMC7271651; doi:10.15252/embr.201949942)
Supplement: Supplementary file 13 — Source Data for Figure 3 [file EMBR-21-e49942-s011.zip › 49942_Fig3_source_data/fig_3_source_data.pptx]

## Slide 1
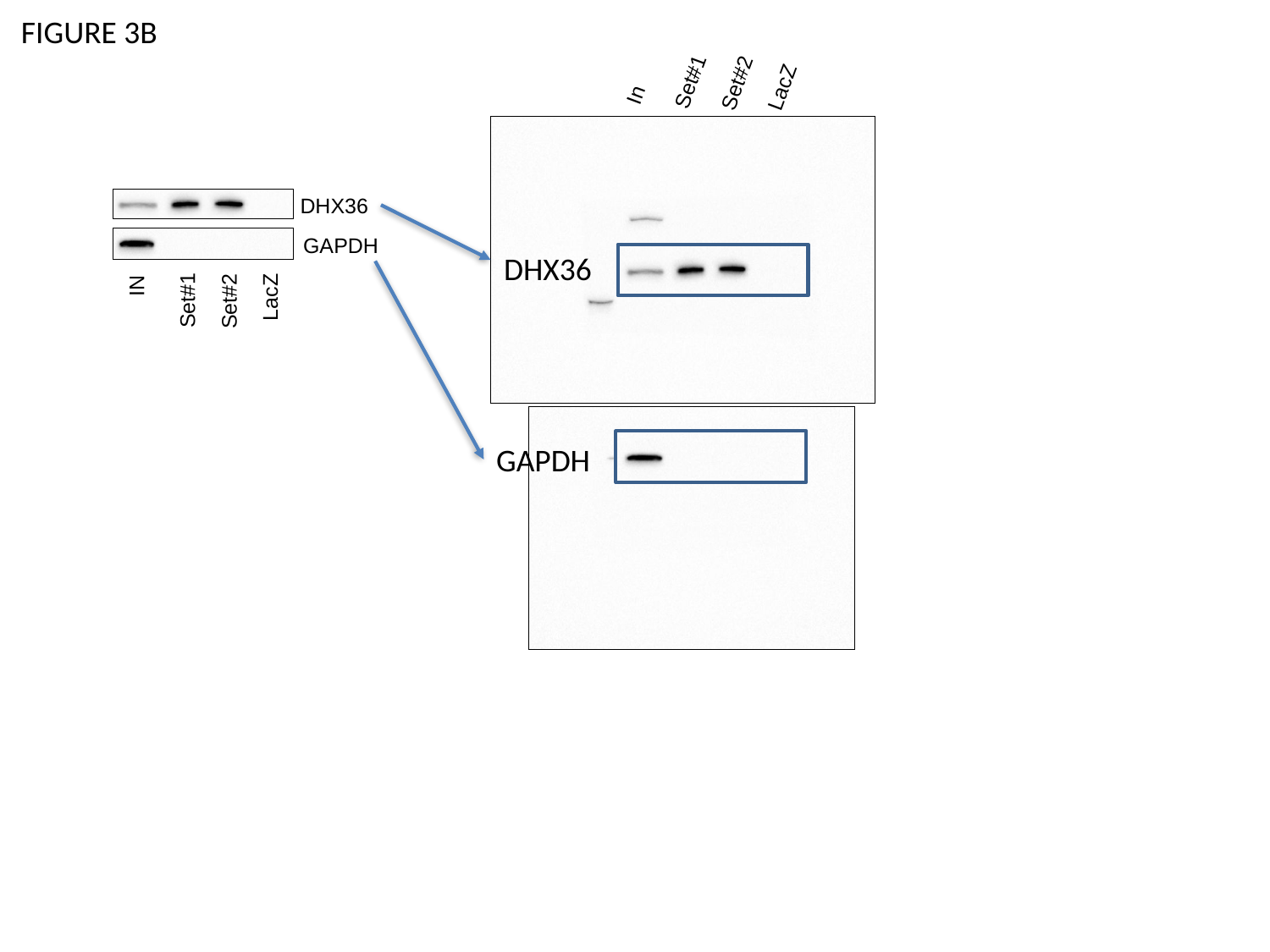

FIGURE 3B
Set#1
In
Set#2
LacZ
DHX36
GAPDH
LacZ
Set#1
Set#2
IN
DHX36
GAPDH

## Slide 2
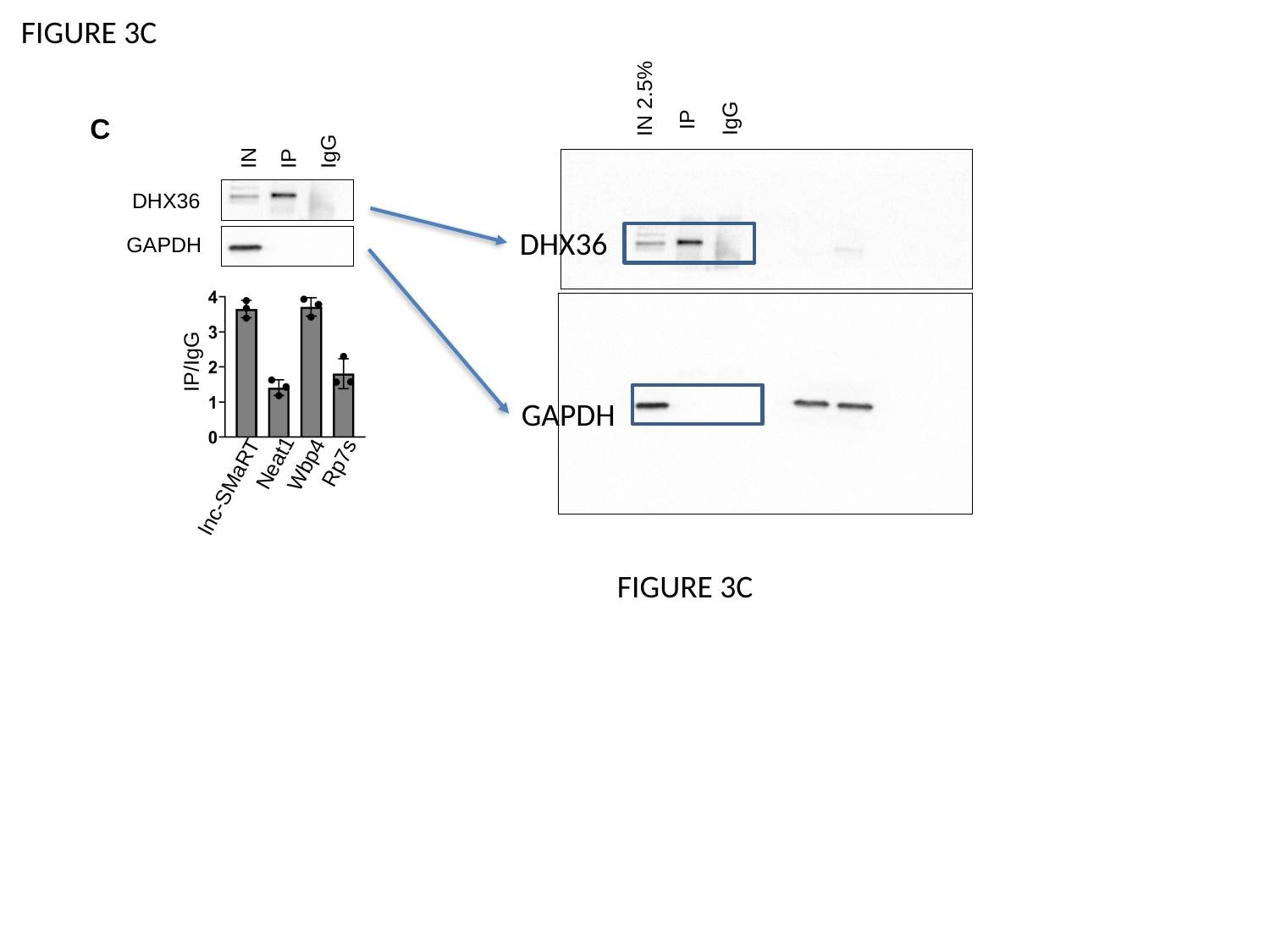

FIGURE 3C
IN 2.5%
IgG
IP
IN
DHX36
GAPDH
IgG
IP
C
DHX36
IP/IgG
Rp7s
Neat1
Wbp4
lnc-SMaRT
GAPDH
FIGURE 3C

## Slide 3
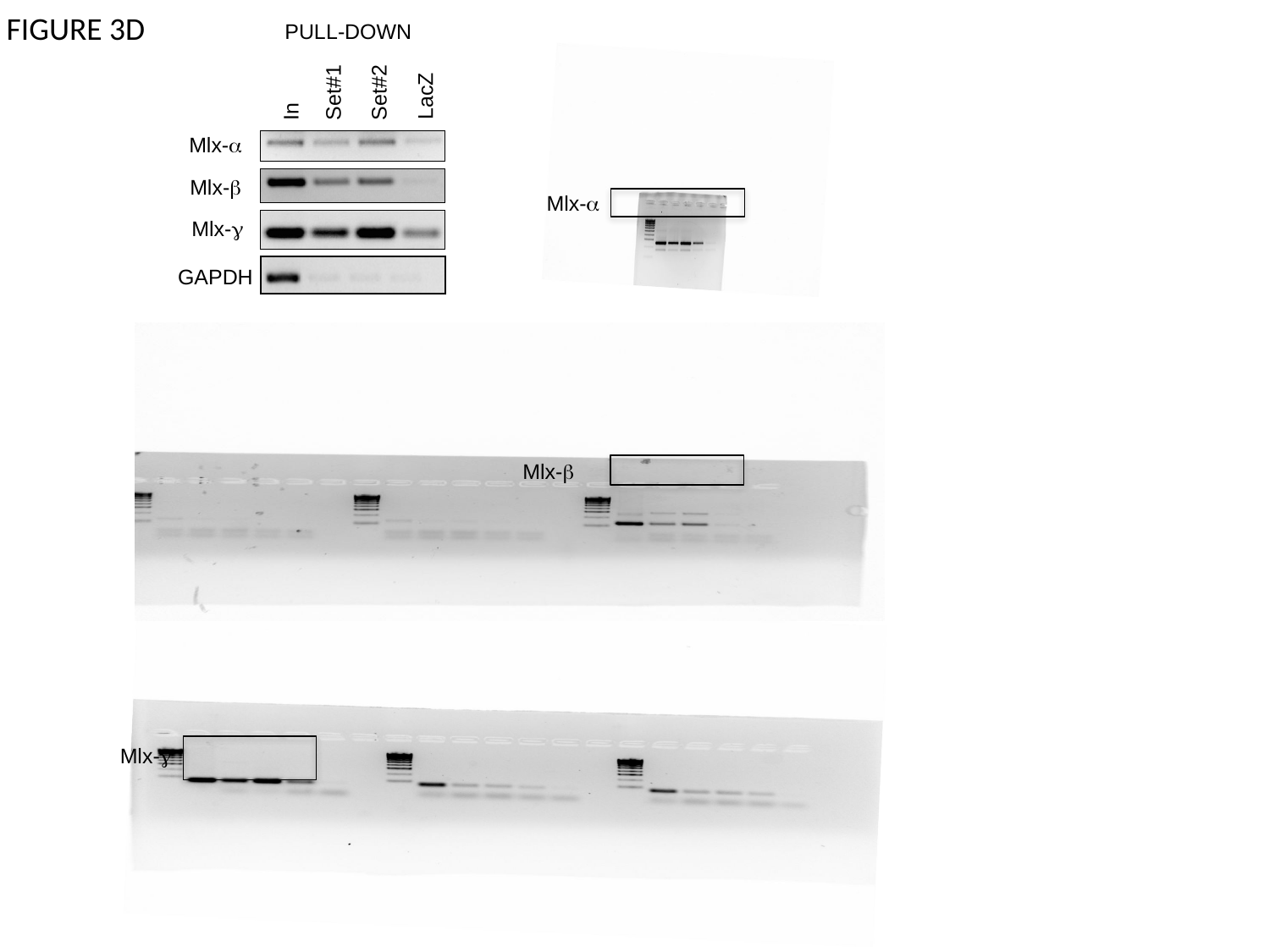

FIGURE 3D
PULL-DOWN
Set#2
Set#1
In
LacZ
Mlx-a
Mlx-b
Mlx-g
GAPDH
Mlx-a
Mlx-b
Mlx-g

## Slide 4
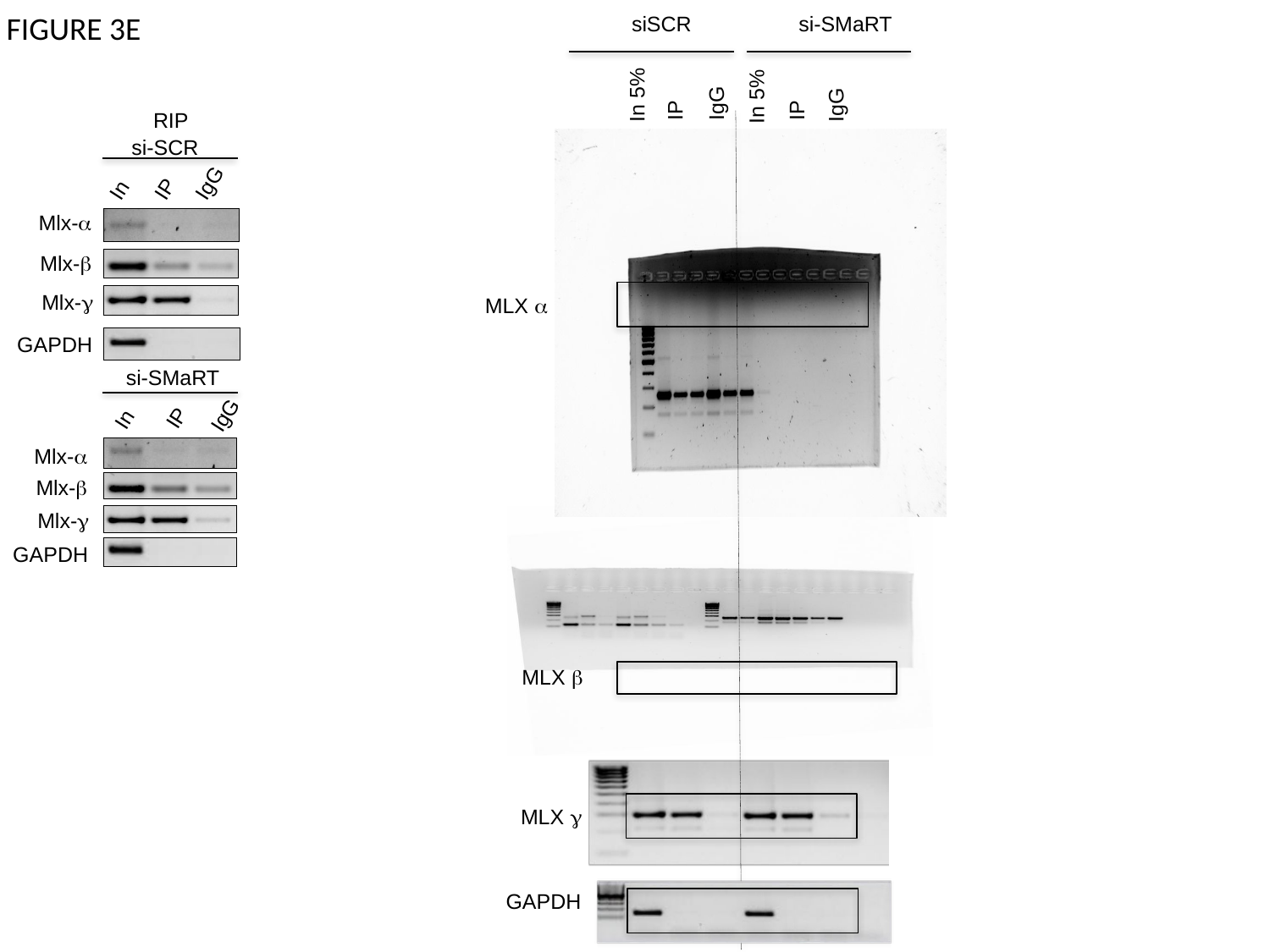

FIGURE 3E
siSCR
si-SMaRT
In 5%
In 5%
IgG
IgG
IP
IP
RIP
si-SCR
In
IP
IgG
Mlx-a
Mlx-b
Mlx-g
MLX a
GAPDH
si-SMaRT
In
IP
IgG
Mlx-a
Mlx-b
Mlx-g
GAPDH
MLX b
MLX g
GAPDH

## Slide 5
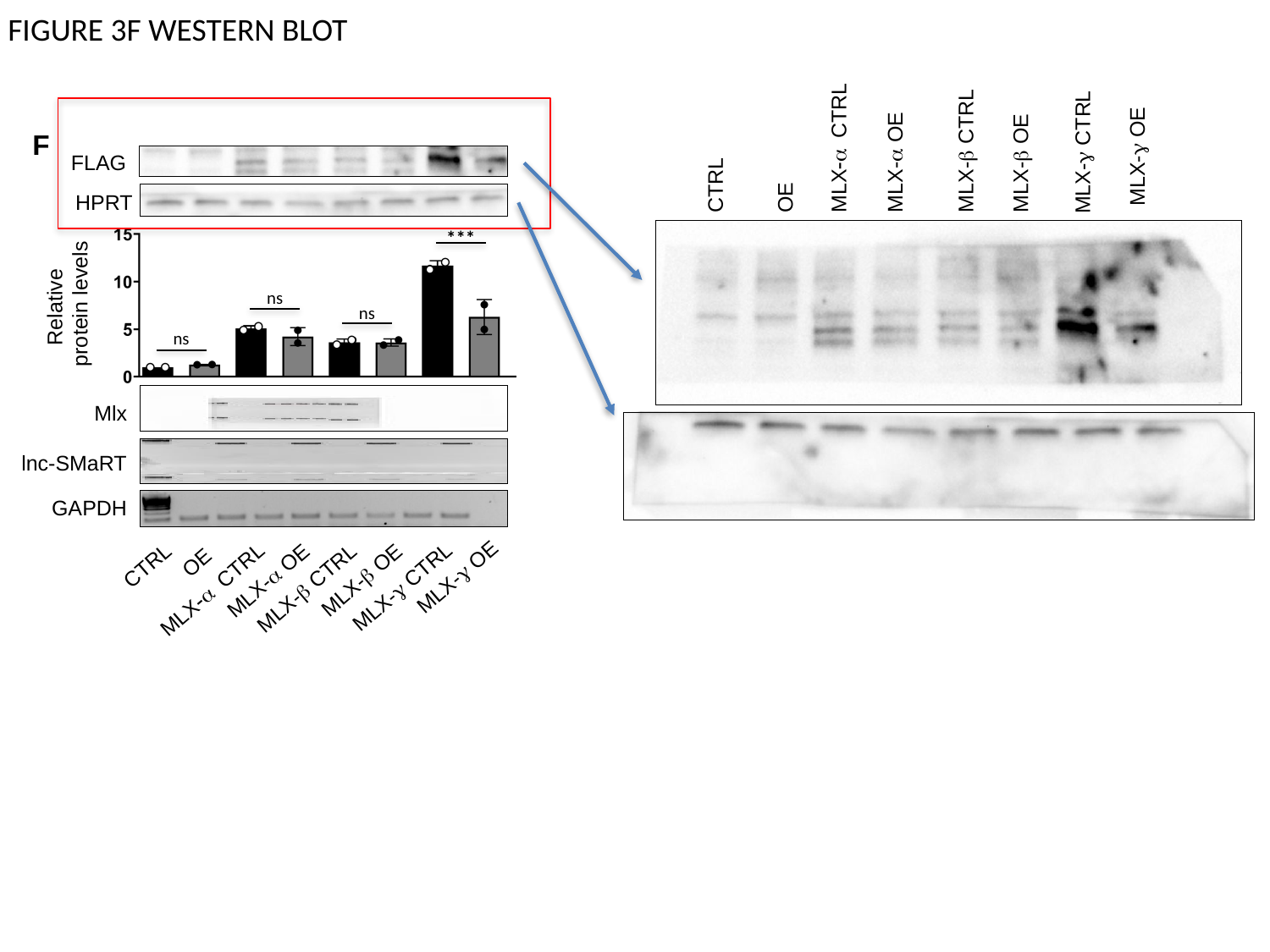

FIGURE 3F WESTERN BLOT
F
FLAG
HPRT
***
Relative
 protein levels
ns
ns
ns
Mlx
lnc-SMaRT
GAPDH
OE
CTRL
MLX-b OE
MLX-a OE
 MLX-g OE
MLX-g CTRL
MLX-b CTRL
MLX-a CTRL
MLX-a CTRL
MLX-b CTRL
MLX-g CTRL
 MLX-g OE
MLX-a OE
MLX-b OE
CTRL
OE

## Slide 6
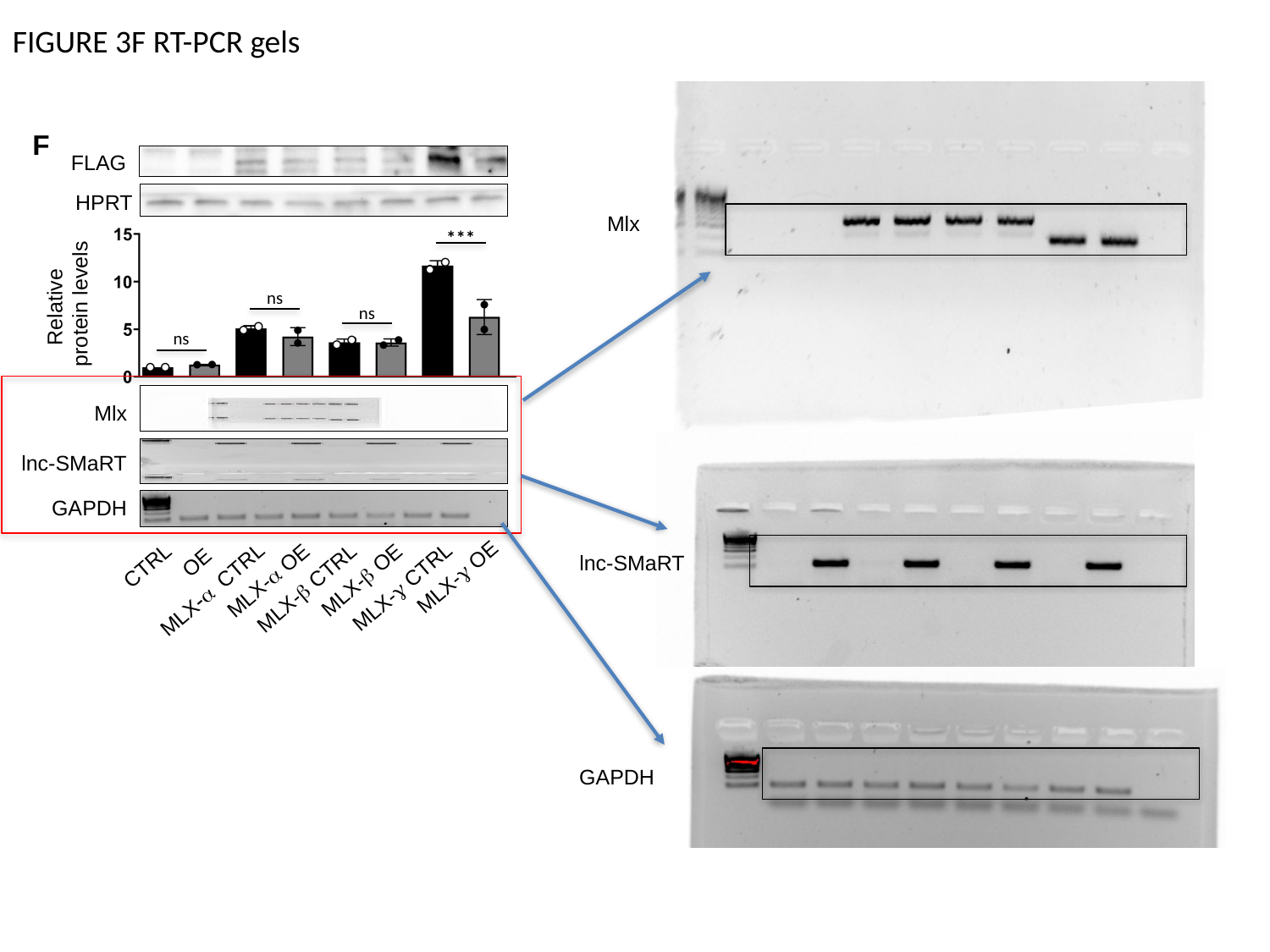

FIGURE 3F RT-PCR gels
F
FLAG
HPRT
***
Relative
 protein levels
ns
ns
ns
Mlx
lnc-SMaRT
GAPDH
OE
CTRL
MLX-b OE
MLX-a OE
 MLX-g OE
MLX-g CTRL
MLX-b CTRL
MLX-a CTRL
Mlx
lnc-SMaRT
GAPDH

## Slide 7
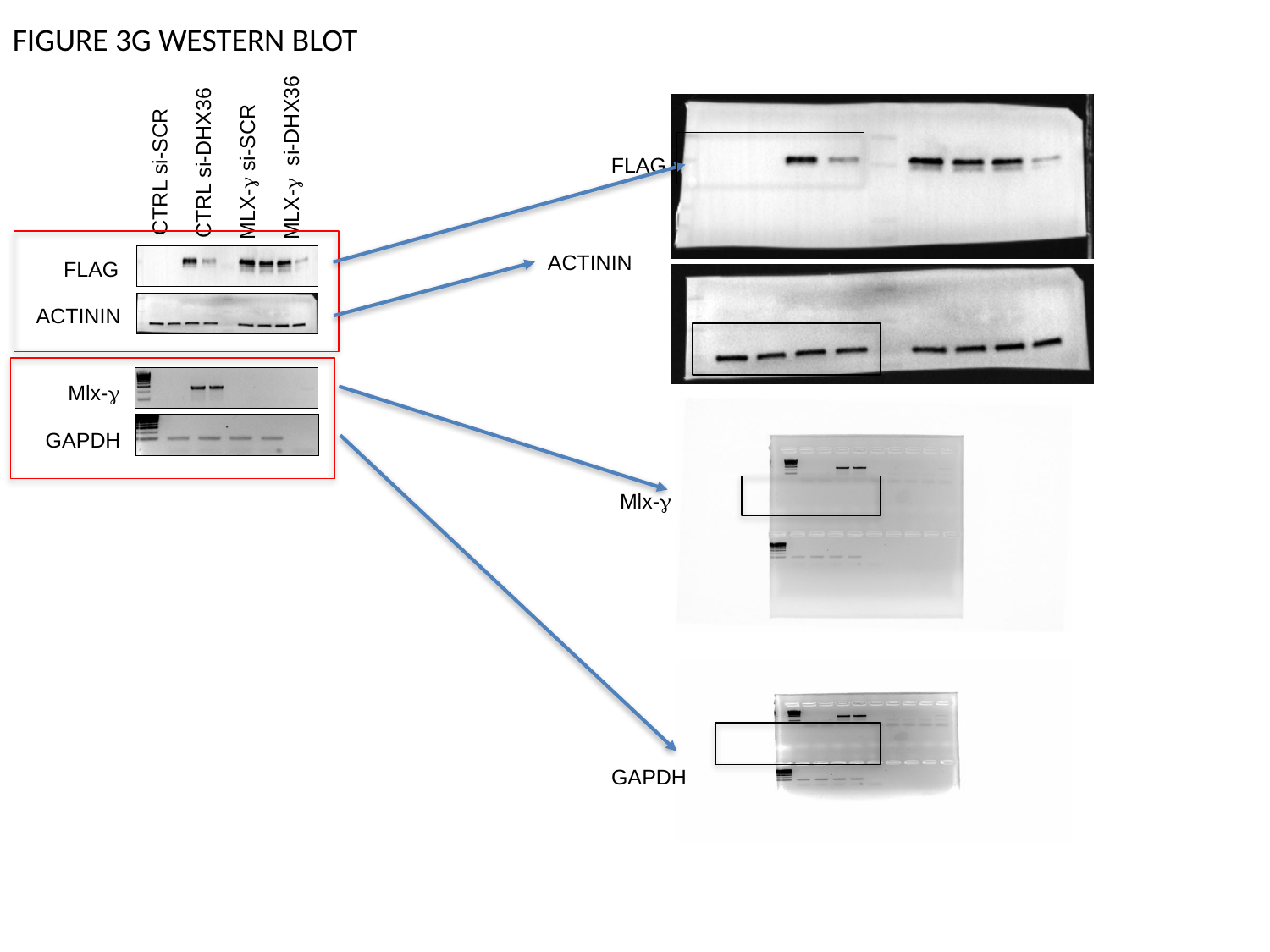

FIGURE 3G WESTERN BLOT
MLX-g si-DHX36
CTRL si-DHX36
CTRL si-SCR
MLX-g si-SCR
FLAG
ACTININ
Mlx-g
GAPDH
FLAG
ACTININ
Mlx-g
GAPDH
